# In silico discovery of 3 novel quercetin derivatives against papain-like protease, spike protein, and 3C-like protease of SARS-CoV-2

**DOI:** 10.1186/s43141-022-00314-7

**Published:** 2022-03-09

**Authors:** Kunal Bhattacharya, Ripunjoy Bordoloi, Nongmaithem Randhoni Chanu, Ramen Kalita, Bhargab Jyoti Sahariah, Atanu Bhattacharjee

**Affiliations:** 1 NETES Institute of Pharmaceutical Science, Mirza, Guwahati, Assam 781125 India; 2 Assam Science and Technology University, Guwahati, Assam India; 3 Faculty of Pharmaceutical Science, Assam Downtown University, Guwahati, Assam India; 4 Royal School of Pharmacy, Royal Global University, Guwahati, Assam India

**Keywords:** 3CLpro, PLpro, Spike protein, Quercetin derivatives, SARS-CoV-2

## Abstract

**Background:**

The derivatives of quercetin is known for their immune-modulating antiviral, anti-blood clotting, antioxidant, and also for its anti-inflammatory efficacy. The current study was therefore conducted to examine the noted novel derivatives of quercetin present in plant sources as an immune modulator and as an antiviral molecule in the COVID-19 disease and also to study their affinity of binding with potential three targets reported for coronavirus, i.e., papain-like protease, spike protein receptor-binding domain, and 3C-like protease.

Based on the high-positive drug-likeness score, the reported derivatives of quercetin obtained from an open-source database were further filtered. Compounds with positive and high drug-likeness scores were further predicted for their potential targets using DIGEP-Pred software, and STRING was used to evaluate the interaction between modulated proteins. The associated pathways were recorded based on the Kyoto Encyclopedia of Genes and Genomes pathway database. Docking was performed finally using PyRx having AutoDock Vina to identify the efficacy of binding between quercetin derivatives with papain-like protease, spike protein receptor-binding domain, and 3C-like protease. The ligand that scored minimum binding energy was chosen to visualize the interaction between protein and ligand. Normal mode analysis in internal coordinates was done with normal mode analysis to evaluate the physical movement and stability of the best protein-ligand complexes using the iMODS server.

**Results:**

Forty bioactive compounds with the highest positive drug-likeness scores were identified. These 40 bioactives were responsible for regulating different pathways associated with antiviral activity and modulation of immunity. Finally, three lead molecules were identified based on the molecular docking and dynamics simulation studies with the highest anti-COVID-19 and immunomodulatory potentials. Standard antiviral drug remdesivir on docking showed a binding affinity of − 5.8 kcal/mol with PLpro, − 6.4 kcal/mol with 3CLpro, and − 8.6 kcal/mol with spike protein receptor-binding domain of SARS-CoV-2, the discovered hit molecules quercetin 3-O-arabinoside 7-O-rhamnoside showed binding affinity of − 8.2 kcal/mol with PLpro, whereas quercetin 3-[rhamnosyl-(1- > 2)-alpha-L-arabinopyranoside] and quercetin-3-neohesperidoside-7-rhamnoside was predicted to have a binding affinity of − 8.5 kcal/mol and − 8.8 kcal/mol with spike protein receptor-binding domain and 3CLpro respectively

**Conclusion:**

Docking study revealed quercetin 3-O-arabinoside 7-O-rhamnoside to possess the highest binding affinity with papain-like protease, quercetin 3-[rhamnosyl-(1- > 2)-alpha-L-arabinopyranoside] with spike protein receptor-binding domain, and quercetin-3-neohesperidoside-7-rhamnoside with 3C-like protease and all the protein-ligand complexes were found to be stable after performing the normal mode analysis of the complexes in internal coordinates.

**Graphical Abstract:**

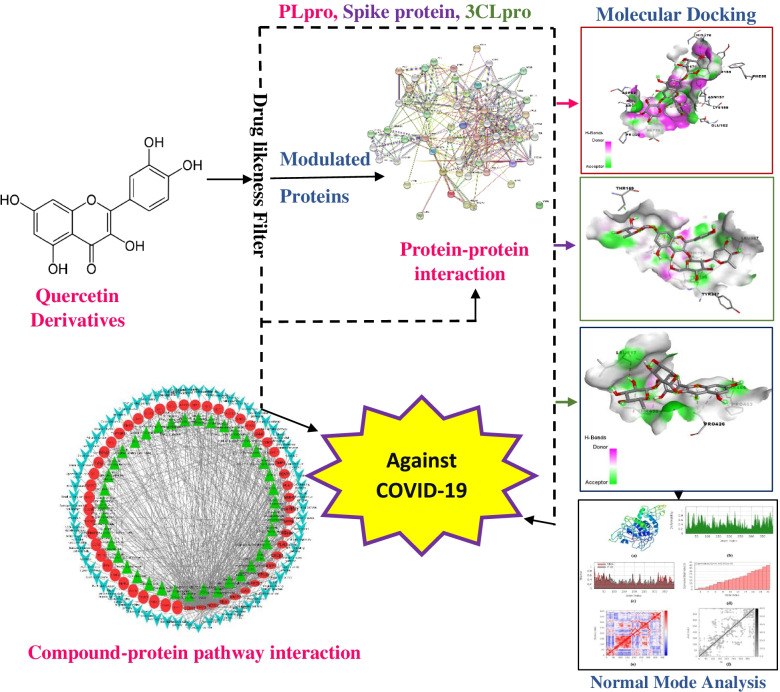

## Background

Starting from 2019 December, now COVID-19 infection caused by the SARS-CoV-2 virus has been the reason for millions of deaths worldwide [1]. People already suffering from different non-infectious and infectious diseases and populations falling under geriatrics and pediatrics having low immunity are at higher risk of getting infected with SARS-CoV-2 induced COVID-19 disease [2]. Different methods such as social distancing and hygiene maintenance, are enforced to tackle the spread of COVID-19; boosting people’s immunity could be of great importance in preventing the spread of the SARS-CoV-2 virus its entry into the body. Although several vaccines have been developed limitations in the manufacturing capacities and distributions capacities, the required herd immunity seems to be a distant mirage. Also, the genetic mutations occurring in the SARS-CoV-2 virus put vaccines’ effectiveness at high risk. To meet the urgent need for a therapeutic agent effective over a broad spectrum of COVID-19 infections, alternate natural source-based anti-covid molecules will be important in fighting COVID-19 disease worldwide. Three potential targets of novel coronaviruses, i.e., papain-like protease (PLpro), spike protein receptor-binding domain, and 3C-like protease (3CLpro) [3–6], are being targeted in search of new lead molecules by the majority of researchers for COVID-19 management. Necrosis of cells and inflammation further worsen the pathogenesis involved in COVID-19, which suggests molecule identification has antiviral, antioxidant, antiviral, immune-modulatory, and anti-inflammatory properties. During the course of its replication, SARS-CoV-2, like all viruses, accumulates mutations—alterations in its genetic code—that make it more dangerous. It is possible that this virus contains built-in RNA repair mechanisms, and as a result, it accumulates mutations at a slower rate than the majority of other RNA viruses. It is estimated that a virus genome from an infection collected in October 2020 has approximately 20 mutations in comparison to the first strain sequenced in January 2020 (Wuhan-Hu-1) [7]. Currently, as of 10th January 2022, as per WHO, four international variants of concern are Beta (B.1.351), first detected in South Africa, Gamma (P.1) first detected in Brazil, Delta (B.1.617.2), first detected in India and Omicron (B.1.1.529) first detected in South Africa and Botswana. The impact on severity was found to be increased in Beta, Gamma, and Delta than the initial variant of SARS-CoV-2 while the impact of Omicron is still unclear [8, 9].

Quercetin derivatives are a group of flavonoids obtained from plants [10]. Quercetin derivatives are chosen particularly for the study because there is substantial evidence in the literature confirming the antiviral activities of quercetin, which has been demonstrated in both in vitro and in vivo tests. In cultured cells, quercetin has been shown to suppress numerous respiratory viruses [11, 12]. Several rhinovirus and echovirus serotypes (types 7, 11, 12, and 19), coxsackievirus (types A21 and B1), and poliovirus (type 1 Sabin) serotypes are inhibited by this compound [13]. Quercetin also has anti-infective and anti-replicative capabilities against RNA and DNA viruses [respiratory syncytial virus (RSV), Polio type 1, parainfluenza type 3, and Herpes simplex virus-1 (HSV-1)] and has been shown to drastically inhibit plaque formation by these viruses [14]. HeLa cells inoculated with cytomegalovirus (CMV) are inhibited in their replication by this compound [15]. Dengue virus type 2 (DENV-2) replication in Vero cells is suppressed by quercetin at an IC50 of 35.7 g/mL, resulting in a 67% drop in DENV-2 RNA levels in the cells. This is due to quercetin’s capacity to either prevent virus entry or suppress replicative enzymes like viral polymerases, which are responsible for virus replication [16]. Quercetin appears to protect mice infected with the meningoencephalitis virus from contracting a deadly illness, according to in vivo research [17]. A positive effect of quercetin administration was also shown in immunocompetent mice infected with the Mengo virus, where it was found to reduce the severity of the organ damage [18]. Athletes who take quercetin supplements are less likely to get an upper respiratory tract infection as a result of stress [19]. Therefore, in COVID-19 disease, it may be fruitful bioactive under investigation, which is identified with antiviral, antioxidant, and anti-inflammatory properties, which can be demonstrated using network pharmacology. Hence, based on the immunity-boosting/anti-inflammatory/anti-viral/antioxidant reports. With the help of in silico molecular docking and various system biology tools, we attempted to evaluate the antiviral efficacy of several derivatives of quercetin.

## Materials and methods

### Bioactive compounds with their drug-likeness score

From the Chemical Entities of Biological Interest (ChEBI) database (https://www.ebi.ac.uk/chebi/).and available records of literature, the phytoconstituents reported under the quercetin phytochemistry were retrieved. For drug-likeness score prediction, all the compounds were screened in MolSoft (https://molsoft.com/mprop/) by querying the SMILES of each molecule.

### Immunity boosting efficacy assessment by target prediction and enrichment analysis

Upregulated and downregulated “protein-based targets” were identified using DIGEP-Pred [20] by querying high-positive drug-likeness scoring derivatives of quercetin at a probable activity of 0.5. The regulated proteins list obtained was further queried using STRING [21]. The probable modulated pathways were also identified using the Kyoto Encyclopedia of Genes and Genomes database. Further, Cytoscape version 3.8.2 was used for network construction between the bioactives, their potential targets, and modulated pathways [22]. To prevent false hit appearance, the elimination of the duplicate interconnection between two nodes was done, and also the entire network was analyzed further using the “network analyzer” tool [23].

### Probable antiviral activity prediction

By keeping pharmacological activity (Pa) > Pharmacological inactivity (Pi), SMILES of each bioactive compound were queried in Prediction of Activity Spectra for Substances using the keyword "antiviral" to get the probable biological spectrum and to predict the antiviral activity of each compound [24]. Further, the records were also queried to identify the possible pharmacological spectrum against different viruses like influenza, herpes, adenovirus, trachoma, hepatitis B, rhinovirus, hepatitis C, cytomegalovirus (CMV), human immunodeficiency virus (HIV) and picornavirus.

### In silico molecular docking

#### Ligand molecules preparation

From the database of PubChem (https://pubchem.ncbi.nlm.nih.gov/) ligands in 3D. sdf format with high positive drug-likeness scores was retrieved, or ChemSketch (https://www.acdlabs.com/resources/freeware/chemsketch/) was used to draw the structures of compounds as applicable. Using Discovery Studio, 2021 [25], all the ligands in .sdf format were converted into .pdb format. UFF was used as a forcefield for energy minimization of the bioactives [26]. After energy minimization, the conversion of all the ligand molecules into. pdbqt format was done.

#### Protein macromolecules preparation

Three potential target proteins of SARS-CoV-2, i.e., PLpro (PDB: 4M0W), spike protein receptor-binding domain (PDB: 6LZG), and 3CLpro (PDB: 6LU7), were selected. Using Discovery studio, 2021, heteroatoms present in the complex with proteins retrieved from Research Collaboratory for Structural Bioinformatics database were removed, and further, the proteins were saved in .pdb format.

#### Ligand-protein docking

Docking was performed between ligand and protein molecules using PyRx having AutoDock vina Plugin [27]. The grid box center values for 4M0W receptor were kept as X:8.6090, Y: 14.6186, and Z: 18.8131, whereas the dimension values in angstrom were X:77.8939, Y:70.8430, Z:25.0000. The grid box center values for 6LU7 receptor were kept as X: -22.9001, Y:14.5229, Z:58.9679, whereas the dimension values in angstrom were X:61.9565, 71.9321, and Z: 25.0000.

The grid box center values for 6LZG receptor were kept as X: -25.7988, Y:18.5947, Z: -25.4521, whereas the dimension values in angstrom were X: 69.3549, Y: 81.5506, and Z:25.0000. By keeping the exhaustiveness value at eight, dockings were performed in order to achieve 9 different ligand molecule poses. After completing docking, the ligand pose gave the minimum binding energy, the value of which was further selected for visualizing the interaction between ligand and protein using Discovery studio 2021 [28, 29].

#### Normal mode analysis in internal coordinates

Normal mode analysis in internal coordinates was carried out for the best ligands among the selected molecules. From the analysis of docking results, it was declared that quercetin 3-O-arabinoside 7-O-rhamnoside was the best ligand for papain-like protease, quercetin 3-[rhamnosyl-(1- > 2)-alpha-L-arabinopyranoside] was the best ligand for spike protein receptor-binding domain, and quercetin-3-neohesperidoside-7-rhamnoside was the best ligand for 3C-like protease. The normal mode analysis for all three protein-ligand complexes was carried out using iMODS server (http://imods.chaconlab.org/). It is a very effective, rapid, and user-friendly tool that can be used for the structural investigation of protein-ligand complexes. The analysis provides deformity values, eigenvalues, B-factor, elastic network details, variance, and covariance map. For a protein-ligand complex, the deformity depends upon the ability to deform at each of its amino acid residues. The energy that is required to deform the structure is understood by eigenvalue, which also represents the motion stiffness of the protein-ligand complex [30, 31].

## Results

### Bioactive compounds and their drug-likeness score

Among 134 quercetin derivatives, 40 bioactives with high drug-likeness scores were identified. Among them, Calabricoside B scored the highest drug-likeness score, i.e., 1.17 with molecular weight 904.23, 23 hydrogen bond acceptor, 13 hydrogen bond donors, and − 1.27 MolLogP. Druglikeness score details of individual compounds are summarized in Table 1.

**Table 1:** Druglikeness of quercetin derivatives with high positive score

Bioactives	Molecular formula	Molecular weight	NHBA	NHBD	MolLogP	MolPSA (A^2^)	MolVol (A^3^)	DLS
Quercetin 3,7-di-*O*-α-L-rhamnoside	C_27_H_30_O_15_	594.16	15	9	− 1.13	195.34	531.93	0.78
Quercetin 3-*O*-rhamnoside-7-*O*-glucoside	C_27_ H_30_ O_16_	610.15	16	10	− 1.99	212.65	539.15	0.78
Quercetin 3-*O*-[β-D-xylosyl-(1→2)-β-D-glucoside]	C_26_ H_28_ O_16_	596.14	16	10	− 1.61	214.98	518.37	0.90
Quercetin 7-*O*-β-L-rhamnopyranoside	C_21_ H_20_ O_11_	448.10	11	7	− 0.26	147.54	406.18	0.83
Quercetin 3-*O*-β-L-fucopyranoside	C_21_ H_20_ O_11_	448.10	11	7	0.32	150.41	407.46	0.82
Quercetin 3-*O*-β-D-glucopyranosyl-7-*O*-α-L-rhamnopyranoside	C_27_ H_30_ O_16_	610.15	16	10	− 1.99	212.65	539.15	0.78
Quercetin 3-*O*-α-(6”'-caffeoylglucosyl-β-1,2-rhamnoside)	C_36_ H_36_ O_19_	772.19	19	11	0.98	250.40	699.90	0.90
Quercetin 3-(6”-*p*-hydroxybenzoylgalactoside)	C_28_ H_24_ O_14_	584.12	14	8	1.40	189.07	524.25	0.83
Quercetin 3-*O*-(2”,3”-digalloyl)-β-D-galactopyranoside	C_35_ H_28_ O_20_	768.12	20	12	1.00	273.40	680.77	1.01
Quercetin 3-*O*-β-D-glucopyranosyl-(1→2)-rhamnopyranoside	C_27_ H_30_ O_16_	610.15	16	10	− 1.15	214.15	533.87	0.88
Quercetin 3-*O*-α-L-[6”'-*p*-coumaroyl-β-D-glucopyranosyl-(1→2)-rhamnopyranoside]	C_36_ H_36_ O_18_	756.19	18	10	1.36	234.92	687.18	0.89
Quercetin 3-*O*-α-L-rhamnopyranosyl-(1→2)-α-L-arabinopyranoside	C_26_ H_26_ O_15_	580.14	15	9	− 0.92	197.67	511.15	1.05
Quercitrin	C_21_ H_20_ O_11_	448.10	11	7	0.32	150.41	407.46	0.82
Quercetin 3-*O*-α-L-[6”'-*p*-coumaroyl-β-D-glucopyranosyl-(1→2)-rhamnopyranoside]-7-*O*-β-D-glucopyranoside	C_42_ H_46_ O_23_	918.24	23	13	− 0.95	297.16	818.87	0.79
Multinoside A	C_27_ H_30_ O_16_	610.15	16	10	− 1.09	214.15	533.87	0.88
Rutin	C_27_ H_30_ O_16_	610.15	16	10	− 1.55	213.63	533.42	0.91
Camellianoside	C_32_ H_38_ O_20_	742.20	20	12	− 2.91	260.89	637.11	1.07
Hermannioside A	C_32_ H_38_ O_19_	726.20	19	11	− 2.37	242.60	635.62	1.01
Quercetin 3'-isobutyrate	C_19_ H_16_ O_8_	372.08	8	4	2.03	106.80	361.56	1.06
Quercetin 3-(2-galloylglucoside)	C_28_ H_24_ O_16_	616.11	16	10	0.29	220.56	547.73	0.97
Quercetin 3-glucoside 7-xyloside	C_26_ H_28_ O_16_	596.14	16	10	− 2.32	213.47	523.65	0.80
Quercetin 3-lathyroside	C_26_ H_28_ O_16_	596.14	16	10	− 1.61	214.98	518.37	0.90
Quercetin 3-(6”-acetylglucoside)	C_23_ H_22_ O_13_	506.11	13	7	0.08	171.83	460.42	0.80
Quercetin 3-(6”-malonylneohesperidoside)	C_30_ H_32_ O_19_	696.15	19	10	− 1.42	245.96	608.70	0.86
Quercetin 3-(2-caffeoylsophoroside) 7-glucoside	C_42_ H_46_ O_25_	950.23	25	15	− 2.54	330.48	838.90	0.87
Quercetin 3-O-arabinoside 7-O-rhamnoside	C_26_ H_28_ O_15_	580.14	15	9	− 1.46	196.16	516.43	0.95
Quercetin 3-(3”,6”-diacetylgalactoside)	C_25_ H_24_ O_14_	548.12	14	6	0.68	176.47	506.25	1.03
Quercetin 3-(2”'-p-coumarylsambubioside)-7-glucoside	C_41_ H_44_ O_23_	904.23	23	13	− 1.91	298.51	803.46	1.02
Quercetin 3-[rhamnosyl-(1- > 2)-alpha-L-arabinopyranoside]	C_26_ H_28_ O_15_	580.14	15	9	− 0.92	197.67	511.15	1.05
Quercetin-7-*O*-[α-L-rhamnopyranosyl(1→6)-β-D-galactopyranoside]	C_27_ H_30_ O_16_	610.15	16	10	− 2.13	210.76	532.15	0.92
Taxifolin	C_15_ H_12_ O_7_	304.06	7	5	0.76	103.49	268.73	1.00
5-galloylquercetin-3-*O*-α-L-arabinofuranoside	C_27_ H_22_ O_14_	570.10	14	9	0.79	198.07	516.59	0.86
Petiolaroside	C_27_ H_30_ O_16_	610.15	16	10	− 1.99	212.65	539.15	0.78
2-(3,4-dihydroxybenzoyloxy)-4,6-dihydroxybenzoic acid	C_14_ H_10_ O_8_	306.04	8	5	1.07	116.37	268.60	1.07
Calabricoside A	C_32_ H_38_ O_20_	742.20	20	12	− 3.23	259.91	642.84	1.00
Calabricoside B	C_41_ H_44_ O_23_	904.23	23	13	− 1.27	296.68	808.96	1.17
Cudranian 2	C_28_ H_26_ O_13_	570.14	13	9	0.74	181.22	516.03	1.06
Quercetin-3'-glucuronide	C_21_ H_20_ O_13_	480.09	13	8	− 0.86	177.94	405.92	1.00
Quercetin-3-*O*-arabinoside	C_20_ H_18_ O_11_	434.08	11	7	− 0.01	151.23	391.96	0.93
Quercetin-3-neohesperidoside-7-rhamnoside	C_33_ H_40_ O_20_	756.21	20	12	− 2.60	259.09	658.34	0.84

*DLS* druglikeness score, *NHBA* number of hydrogen bond acceptor, *NHBD* number of hydrogen bond donor

### Target prediction and their enrichment analysis to assess immune-boosting efficacy

**Fig. 1:**
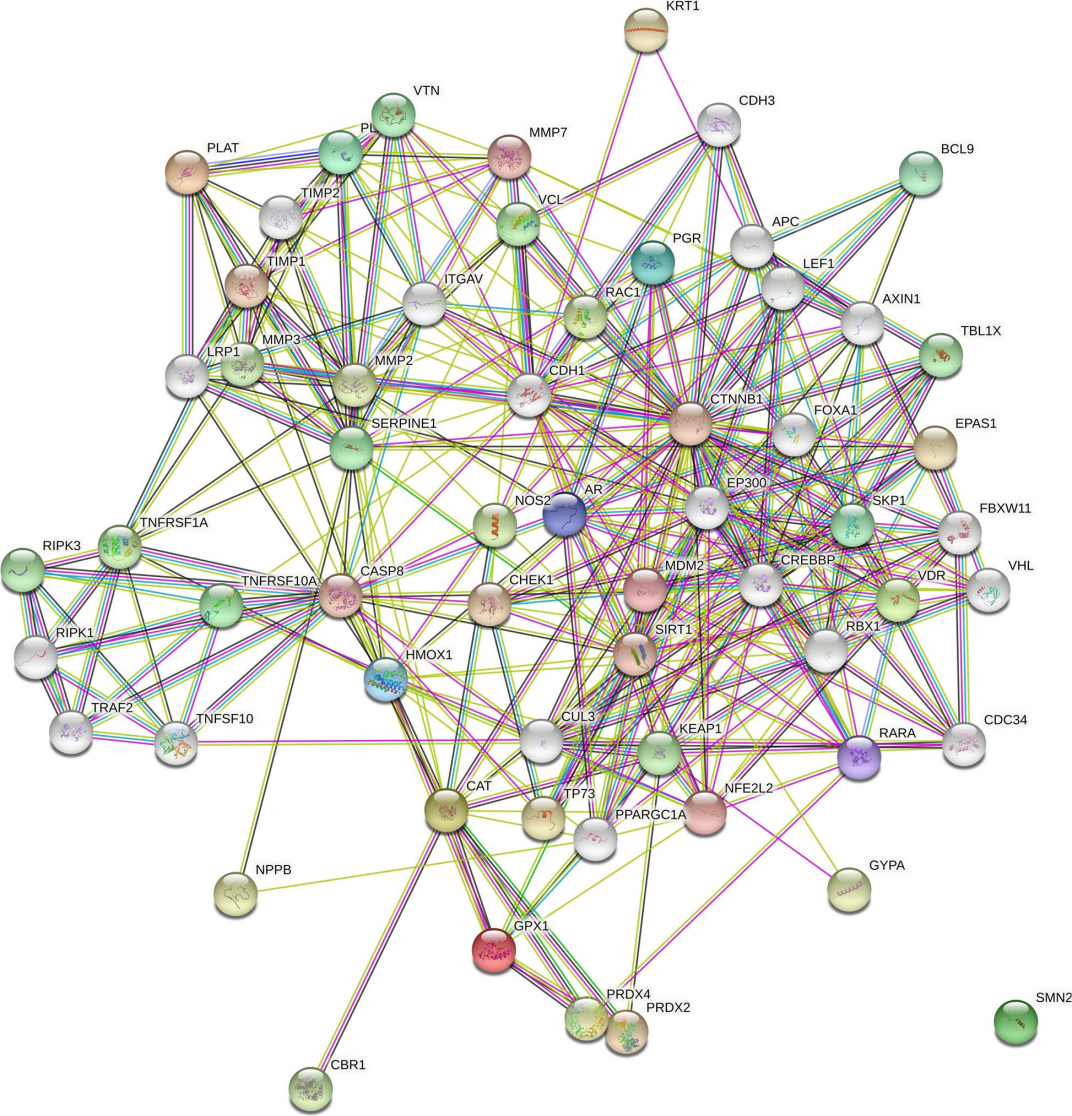
Protein-protein interaction of regulated proteins

**Fig. 2:**
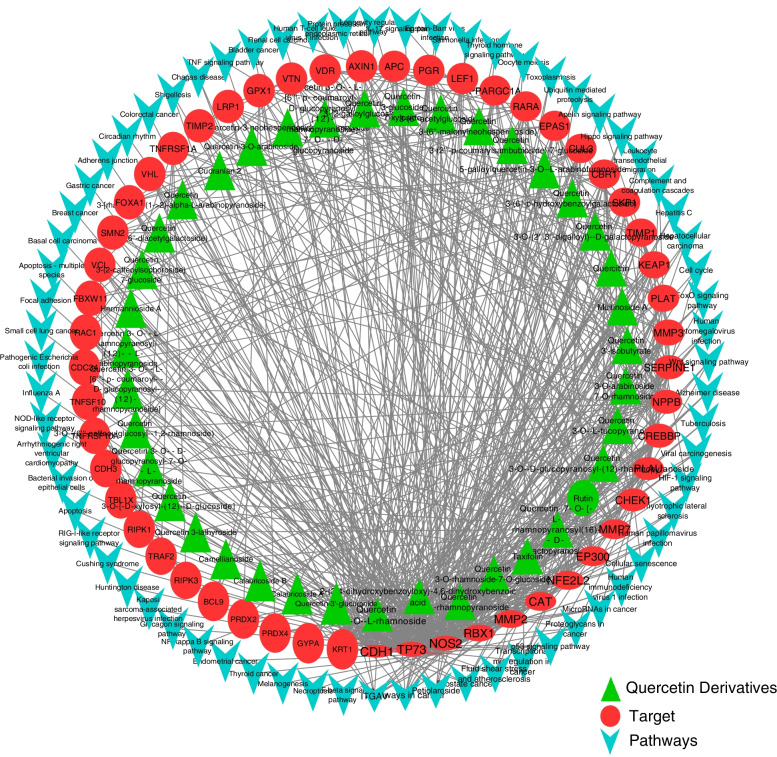
Network interaction of quercetin derivatives with their proteins and regulated pathways

**Fig. 3:**
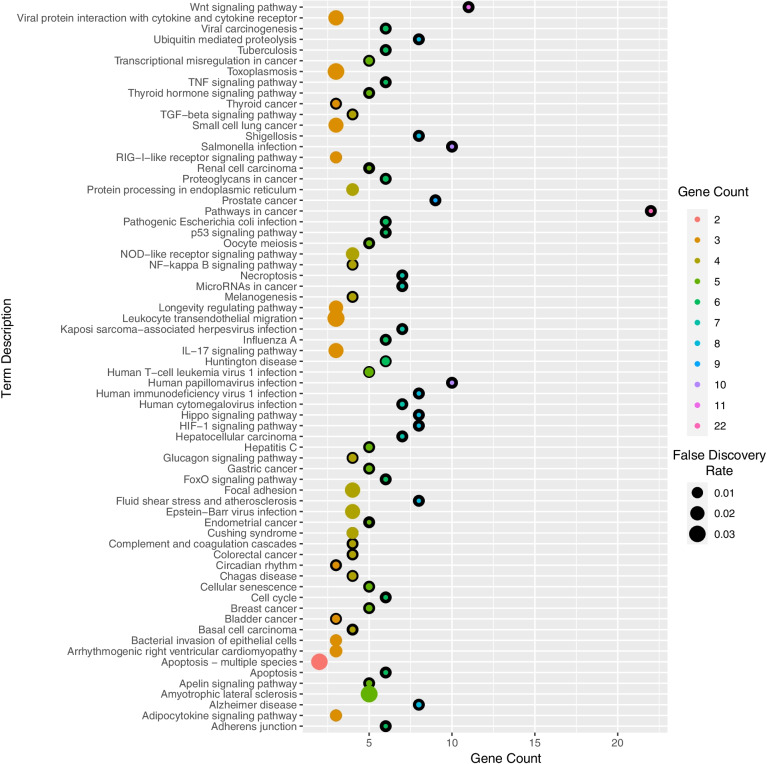
Dot Plot of KEGG pathway enrichment analysis

Among all the compounds having a high-positive drug-likeness score, it was predicted that quercetin 3,7-di-O-α-L-rhamnoside modulates the maximum number of genes, i.e., 10. Also, Cadherin-1 (CDH-1) was targeted by the maximum number of bioactive compounds, i.e., 30. Further, 61 different pathways were identified by enrichment analysis in which cancer pathways were majorly modulated via 22 genes (KEAP1, HMOX1, RBX1, MMP2, SKP1, TRAF2, RARA, VHL, APC, MDM2, ITGAV, CDH1, AXIN1, CREBBP, EP300 EPAS1, LEF1, NOS2, CTNNB1, CASP8, AR, NFE2L2) under the background of 517 proteins at the false discovery rate of 7.71E−17. Modulated gene set's enrichment analysis with its modulated pathway and individual gene codes is summarized in Table 2. The protein-protein interaction of the modulated proteins is given in Fig. 1. The combined bioactive-proteins-pathways is given in Fig. 2. which also reflected the quercetin 3,7-di-O-α-L-rhamnoside to target the maximum number of proteins. The dot plot for KEGG Pathway analysis is given in Fig. 3

**Table 2:** Enrichment analysis of modulated proteins by the reported quercetin derivatives

Term ID	Term description	Observed gene count	Back-gene count	False discovery rate	Matching proteins in the network
hsa05200	Pathways in cancer	22	517	7.71E−17	KEAP1,HMOX1,RBX1,MMP2,SKP1,TRAF2,RARA,VHL,APC,MDM2,ITGAV,CDH1,AXIN1,CREBBP,EP300,EPAS1,LEF1,NOS2,CTNNB1,CASP8,AR,NFE2L2
hsa04310	Wnt signalling pathway	11	154	5.78E−10	RBX1,TBL1X,SKP1,APC,MMP7,A IN1,CREBBP,EP300,FBXW11,LEF CTNNB1
hsa05215	Prostate cancer	9	96	4.52E−09	PLAT,MDM2,CREBBP,EP300,LEF1, MP3,CTNNB1,PLAU,AR
hsa05132	Salmonella infection	10	209	1.28E−07	TNFRSF1A,RIPK3,TNFRSF10A,SKP1,TNFSF10,TRAF2,RIPK1,LEF1,CTNNB1,CASP8
hsa04066	HIF-1 signaling pathway	8	106	1.83E−07	HMOX1,RBX1,TIMP1,SERPINE1,VHL,CREBBP,EP300,NOS2
hsa05418	Fluid shear stress and atherosclerosis	8	130	6.93E−07	TNFRSF1A,KEAP1,HMOX1,M P2,PLAT,ITGAV,CTNNB1,NFE L2
hsa04120	Ubiquitin mediated proteolysis	8	135	7.86E−07	KEAP1,CDC34,RBX1,SKP1,VHL,MDM2,CUL3,FBXW11
hsa04390	Hippo signaling pathway	8	153	1.74E−06	SERPINE1,APC,CDH1,AXIN1,FBXW11,LEF1,CTNNB1,TP73
hsa05165	Human papillomavirus infection	10	325	3.28E−06	TNFRSF1A,VTN,APC,MDM2,ITGAV,AXIN1,CREBBP,EP300,CTNNB1,CASP8
hsa04520	Adherens junction	6	67	4.16E−06	VCL,CDH1,CREBBP,EP300,LEF1,CTNNB1
hsa04115	p53 signaling pathway	6	72	5.65E−06	TNFRSF10A,SERPINE1,MDM2,CASP8,TP73,CHEK1
hsa05170	Human immunodeficiency virus 1 infection	8	204	9.71E−06	TNFRSF1A,RBX1,SKP1,TRAF2,RIPK1,FBXW11,CASP8,CHEK1
hsa05131	Shigellosis	8	218	1.46E−05	TNFRSF1A,VCL,RBX1,SKP1,TRAF2,MDM2,RIPK1,FBXW11
hsa04217	Necroptosis	7	149	1.54E−05	TNFRSF1A,RIPK3,TNFRSF10A,TNFSF10,TRAF2,RIPK1,CASP8
hsa05206	MicroRNAs in cancer	7	160	2.28E−05	SIRT1,HMOX1,APC,MDM2,CREBBP,EP300,PLAU
hsa05225	Hepatocellular carcinoma	7	160	2.28E−05	KEAP1,HMOX1,APC,AXIN1,LEF1,CTNNB1,NFE2L2
hsa05213	Endometrial cancer	5	57	3.41E−05	APC,CDH1,AXIN1,LEF1,CTNNB1
hsa04668	TNF signaling pathway	6	112	4.03E−05	TNFRSF1A,RIPK3,TRAF2,RIPK1,MMP3,CASP8
hsa05167	Kaposi sarcoma-associated herpesvirus infection	7	187	4.90E−05	TNFRSF1A,TRAF2,CREBBP,EP300,LEF1,CTNNB1,CASP8
hsa04110	Cell cycle	6	120	5.32E−05	RBX1,SKP1,MDM2,CREBBP,EP300,CHEK1
hsa05211	Renal cell carcinoma	5	66	5.43E−05	RBX1,VHL,CREBBP,EP300,EPAS1
hsa04068	FoxO signaling pathway	6	127	6.62E−05	SIRT1,CAT,TNFSF10,MDM2,CREBBP,EP300
hsa04210	Apoptosis	6	132	7.84E−05	TNFRSF1A,TNFRSF10A,TNFSF10,TRAF2,RIPK1,CASP8
hsa05163	Human cytomegalovirus infection	7	218	0.0001	TNFRSF1A,TRAF2,MDM2,RIPK1,ITGAV,CTNNB1,CASP8
hsa05010	Alzheimer disease	8	355	0.00025	TNFRSF1A,LRP1,TRAF2,APC,AXIN1,NOS2,CTNNB1,CASP8
hsa05152	Tuberculosis	6	168	0.00025	TNFRSF1A,CREBBP,EP300,NOS2,CASP8,VDR
hsa05164	Influenza A	6	165	0.00025	TNFRSF1A,TNFRSF10A,TNFSF10,CREBBP,EP300,CASP8
hsa05203	Viral carcinogenesis	6	182	0.00038	TRAF2,MDM2,CREBBP,EP300,CASP8,CHEK1
hsa05130	Pathogenic Escherichia coli infection	6	187	0.00042	TNFRSF1A,TNFRSF10A,TNFSF10,TRAF2,RIPK1,CASP8
hsa05205	Proteoglycans in cancer	6	196	0.00053	MMP2,VTN,MDM2,ITGAV,CTNNB1,PLAU
hsa04919	Thyroid hormone signaling pathway	5	119	0.00056	MDM2,ITGAV,CREBBP,EP300,CTNNB1
hsa04114	Oocyte meiosis	5	120	0.00057	RBX1,SKP1,FBXW11,PGR,AR
hsa05217	Basal cell carcinoma	4	62	0.00068	APC,AXIN1,LEF1,CTNNB1
hsa04371	Apelin signaling pathway	5	131	0.0008	PLAT,SERPINE1,CDH1,PPARGC1A,NOS2
hsa05224	Breast cancer	5	145	0.0012	APC,AXIN1,LEF1,PGR,CTNNB1
hsa05226	Gastric cancer	5	144	0.0012	APC,CDH1,AXIN1,LEF1,CTNNB1
hsa04218	Cellular senescence	5	150	0.0014	SIRT1,SERPINE1,MDM2,FBXW11,CHEK1
hsa04610	Complement and coagulation cascades	4	82	0.0016	PLAT,SERPINE1,VTN,PLAU
hsa04710	Circadian rhythm	3	30	0.0016	RBX1,SKP1,FBXW11
hsa05160	Hepatitis C	5	156	0.0016	TNFRSF1A,TRAF2,RIPK1,CTNNB1,CASP8
hsa05210	Colorectal cancer	4	82	0.0016	APC,AXIN1,LEF1,CTNNB1
hsa04350	TGF-beta signaling pathway	4	91	0.0022	RBX1,SKP1,CREBBP,EP300
hsa05202	Transcriptional misregulation in cancer	5	171	0.0022	PLAT,RARA,MDM2,MMP3,PLAU
hsa04916	Melanogenesis	4	95	0.0024	CREBBP,EP300,LEF1,CTNNB1
hsa05216	Thyroid cancer	3	36	0.0024	CDH1,LEF1,CTNNB1
hsa05142	Chagas disease	4	99	0.0027	TNFRSF1A,SERPINE1,NOS2,CASP8
hsa04064	NF-kappa B signaling pathway	4	101	0.0029	TNFRSF1A,TRAF2,RIPK1,PLAU
hsa04922	Glucagon signaling pathway	4	101	0.0029	SIRT1,CREBBP,EP300,PPARGC1A
hsa05016	Huntington disease	6	298	0.0030	TRAF2,CREBBP,EP300,PPARGC1A,CASP8,GPX1
hsa05219	Bladder cancer	3	41	0.0030	MMP2,MDM2,CDH1
hsa05166	Human T-cell leukemia virus 1 infection	5	211	0.0046	TNFRSF1A,MMP7,CREBBP,EP300,CHEK1
hsa04934	Cushing syndrome	4	153	0.0118	APC,AXIN1,LEF1,CTNNB1
hsa04920	Adipocytokine signaling pathway	3	69	0.0119	TNFRSF1A,TRAF2,PPARGC1A
hsa04622	RIG-I-like receptor signaling pathway	3	70	0.0122	TRAF2,RIPK1,CASP8
hsa05100	Bacterial invasion of epithelial cells	3	70	0.0122	VCL,CDH1,CTNNB1
hsa04141	Protein processing in endoplasmic reticulum	4	165	0.0144	RBX1,SKP1,TRAF2,NFE2L2
hsa05412	Arrhythmogenic right ventricular cardiomyopathy	3	76	0.0145	ITGAV,LEF1,CTNNB1
hsa04621	NOD-like receptor signaling pathway	4	174	0.0168	RIPK3,TRAF2,RIPK1,CASP8
hsa04211	Longevity regulating pathway	3	87	0.0203	SIRT1,CAT,PPARGC1A
hsa04657	IL-17 signaling pathway	3	92	0.0233	TRAF2,MMP3,CASP8
hsa05169	Epstein-Barr virus infection	4	193	0.0233	TRAF2,MDM2,RIPK1,CASP8
hsa05222	Small cell lung cancer	3	92	0.0233	TRAF2,ITGAV,NOS2
hsa04510	Focal adhesion	4	198	0.0244	VCL,VTN,ITGAV,CTNNB1
hsa04061	Viral protein interaction with cytokine and cytokine receptor	3	96	0.0245	TNFRSF1A,TNFRSF10A,TNFSF10
hsa04215	Apoptosis - multiple species	2	30	0.0300	TNFRSF1A,CASP8
hsa05145	Toxoplasmosis	3	105	0.0304	TNFRSF1A,NOS2,CASP8
hsa05014	Amyotrophic lateral sclerosis	5	352	0.0316	TNFRSF1A,CAT,TRAF2,NOS2,GPX1
hsa04670	Leukocyte transendothelial migration	3	109	0.0326	VCL,MMP2,CTNNB1

### Possible antiviral activity prediction

**Fig. 4:**
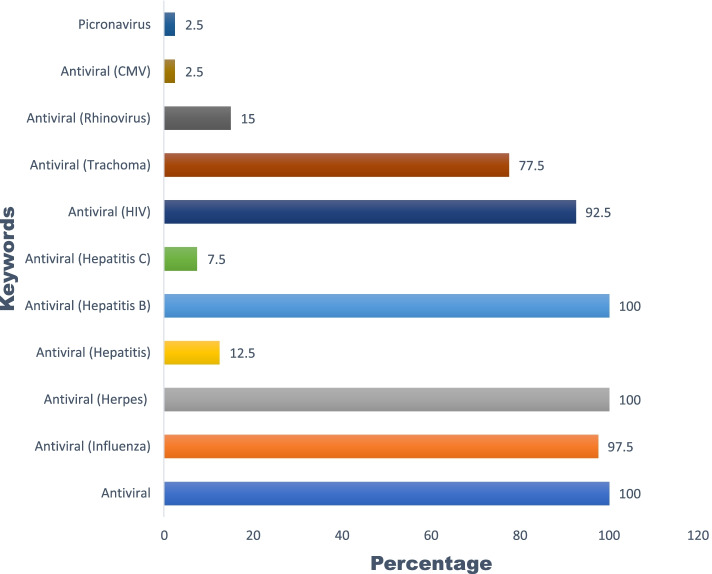
Predicted antiviral activity of quercetin derivatives against multiple viruses

The quercetin derivatives were found to have antiviral potential against influenza, herpes, hepatitis, hepatitis B, hepatitis C, rhinovirus, HIV, CMV, trachoma, and picornavirus. Among them, the maximum number of the compounds were active against herpes virus and hepatitis B virus, i.e., 100%. The overall activity of bioactive compounds against different viruses is given in Fig. 4.

### In silico molecular docking

**Fig. 5:**
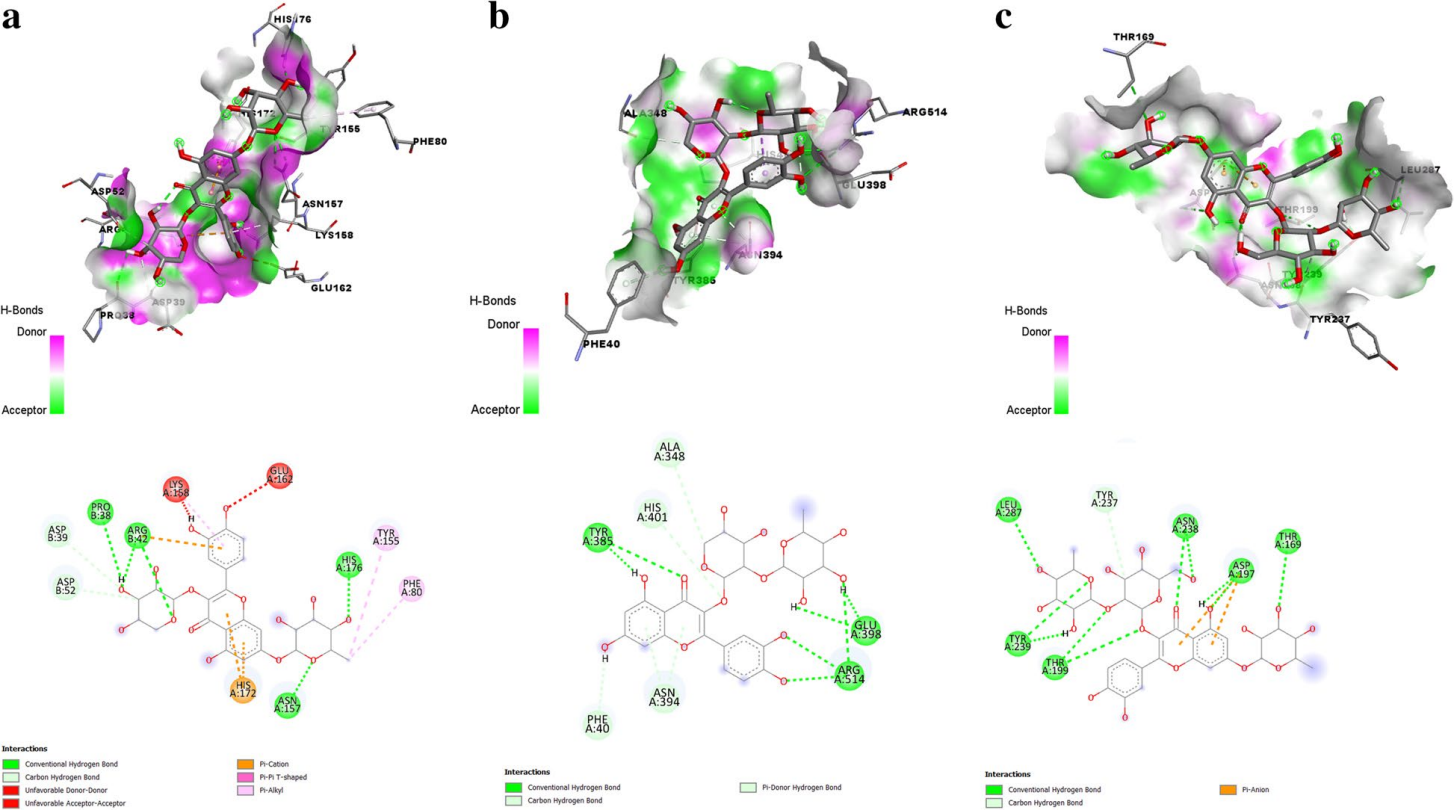
Interaction of **a** Quercetin 3-O-arabinoside 7-O-rhamnoside with papain-like protease. **b** Quercetin 3-[rhamnosyl-(1- > 2)-alpha-L-arabinopyranoside] with spike protein receptor binding domain. **c** Quercetin-3-neohesperidoside-7-rhamnoside with 3C-like protease

**Fig. 6:**
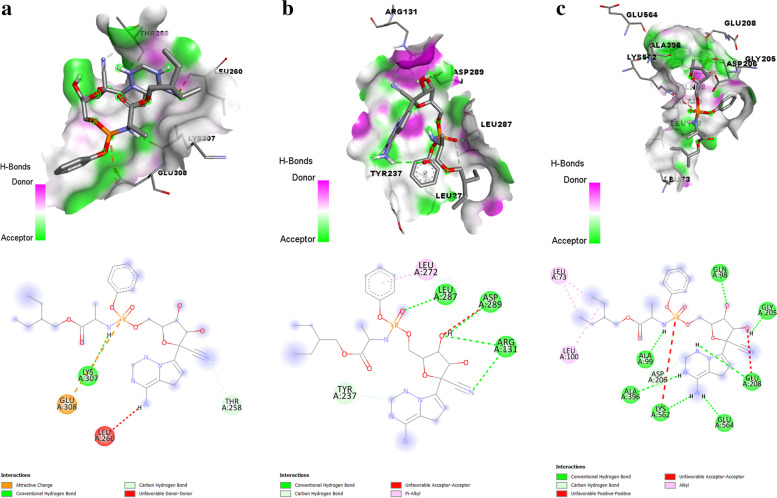
Interaction of **a** Remdesivir with PLpro. **b** Remdesivir with 3CLpro. **c** Remdesivir with spike protein receptor binding domain

Quercetin 3-O-arabinoside 7-O-rhamnoside was speculated to have the maximum binding affinity (-8.2 kcal/mol) with PLpro with 9 hydrogen bond interactions via LYS27B, GLN41B, ARG42B, ARG72B, ARG74B, ASN157A, LYS158A, GLU162A, and HIS176A. The interaction details obtained using PLIP (https://plip-tool.biotec.tu-dresden.de/plip-web/plip/index) [32] are summarized in Table 3. Quercetin 3-[rhamnosyl-(1- > 2)-alpha-L-arabinopyranoside] was speculated in possessing maximum binding affinity (− 8.5 kcal/mol) with spike protein receptor-binding domain with 7 hydrogen bond interaction via ALA348A, TYR385A, ASN394, GLU398A, ARG514A. The interaction details obtained using PLIP (https://plip-tool.biotec.tu-dresden.de/plip-web/plip/index) [32] are summarized in Table 4. Quercetin-3-neohesperidoside-7-rhamnoside was predicted to possess maximum binding affinity (− 8.8 kcal/mol) with 3CLpro with 10 hydrogen bond interactions via ASN133A, THR169A, ALA194A, ASP197A, THR199A, ASN238A, and LEU287A. The interaction details obtained using PLIP (https://plip-tool.biotec.tu-dresden.de/plip-web/plip/index) [32] are summarized in Table 5. The interaction of quercetin 3-O-arabinoside 7-O-rhamnoside with PLpro, quercetin 3-[rhamnosyl-(1- > 2)-alpha-L-arabinopyranoside] with spike protein receptor-binding domain, and quercetin-3-neohesperidoside-7-rhamnoside with 3CLpro is shown in Fig. 5. Remdesivir as a standard antiviral drug showed a binding affinity of − 5.8 kcal/mol with PLpro, − 6.4 kcal/mol with 3CLpro, and − 8.6 kcal/mol with spike protein receptor-binding domain of SARS-CoV-2. 2D results of amino acid residues of protein-ligand interactions showed that remdesivir interacted with LYS A:307, GLU A:308, THR A:258, and LEU A:260 of PLpro, LEU A:272, LEU A:287, ASP A:289, ARG A:131 and TYR A:237 of 3CLpro and LEU A:73, LEU A:100, ALA A:99, ALA A:396, ASP A:206, LYS A:562, GLU A:564, GLU A:208, GLY A:205, and GLN A:98 of spike protein receptor-binding domain of SARS-CoV-2 (Fig. 6).

**Table 3:** Interactions of quercetin 3-O-arabinoside 7-O-rhamnoside with papain-like protease

Hydrogen bonds							
Index	Residue	AA	Distance H-A	Distance D-A	Donor angle	Donor atom	Acceptor atom
1	27B	LYS	2.12	2.80	122.17	3928 [O3]	3532 [O2]
2	41B	GLN	3.45	3.93	112.72	3551 [Ng+]	3928 [O3]
3	42B	ARG	2.27	2.90	119.01	3852 [Ng+]	3915 [O3]
4	72B	ARG	3.37	3.79	107.06	3878 [Ng+]	3915 [O3]
5	74B	ARG	3.26	4.01	131.04	1521 [Nam]	3938 [O3]
6	157A	ASN	2.43	2.86	104.85	1534 [N3+]	3917 [O3]
7	158A	LYS	2.04	2.94	145.30	1568 [O3]	3915 [O3]
8	162A	GLU	2.09	2.70	121.02	1568 [O3]	3915 [O3]
9	176A	HIS	2.10	3.06	154.95	1726 [Npl]	3944 [O3]
Hydrophobic interactions							
Index	Residue	AA	Distance	Ligand Atom	Protein atom		
1	157A	ASN	3.92	3907	1519		
Salt bridges							
Index	Residue	AA	Distance	Ligand Group	Ligand atoms		
1	27B	LYS	5.23	Carboxylate	3921,3926		
2	42B	ARG	4.09	Carboxylate	3919,3921		
3	42B	ARG	4.86	Carboxylate	3921,3926		
4	172A	HIS	4.56	Carboxylate	3898,3944

**Table 4:** Interactions of quercetin 3-[rhamnosyl-(1- > 2)-alpha-L-arabinopyranoside] with spike protein receptor-binding domain

Hydrogen bonds							
Index	Residue	AA	Distance H-A	Distance D-A	Donor angle	Donor atom	Acceptor atom
1	348A	ALA	2.66	3.55	150.52	2678 [Nam]	6459 [O3]
2	385A	TYR	2.11	2.70	119.95	6461 [O3]	2987 [O3]
3	394A	ASN	2.62	3.40	136.26	3058 [Nam]	6415 [O3]
4	398A	GLU	1.81	2.76	163.63	6453 [O3]	3080 [O2]
5	398A	GLU	2.33	3.01	126.77	6451 [O3]	3080 [O2]
6	514A	ARG	2.35	3.19	145.13	6455 [O3]	4037 [Ng+]
7	514A	ARG	2.79	3.55	134.11	4040 [Ng+]	6453 [O3]
Salt bridges							
Index	Residue	AA	Distance	Ligand group	Ligand atoms		
1	401A	HIS	5.25	Carboxylate	6443,6451

**Table 5:** Interactions of quercetin-3-neohesperidoside-7-rhamnoside with 3C-like protease

Hydrogen bonds							
Index	Residue	AA	Distance H-A	Distance D-A	Donor angle	Donor atom	Acceptor atom
1	133A	ASN	2.53	3.41	149.00	1031 [Nam]	2432 [O3]
2	169A	THR	2.36	2.99	121.92	2430 [O3]	1311 [O3]
3	194A	ALA	3.28	3.68	106.41	1492 [Nam]	2428 [O3]
4	194A	ALA	2.21	2.77	115.85	2428 [O3]	1495 [O2]
5	197A	ASP	2.71	3.33	121.36	1508 [Nam]	2418 [O3]
6	197A	ASP	3.58	4.09	115.72	2432 [O3]	1515 [O-]
7	199A	THR	2.65	3.24	119.32	2399 [O3]	1529 [O3]
8	199A	THR	2.32	3.05	131.84	1529 [O3]	2387 [O3]
9	238A	ASN	3.11	4.09	172.34	1852 [Nam]	2386 [O3]
10	287A	LEU	2.29	3.26	166.57	2206 [Nam]	2395 [O3]
Hydrophobic interactions							
Index	Residue	AA	Distance	Ligand atom	Protein atom		
1	197A	ASP	3.41	2378	1512		
2	287A	LEU	3.70	2392	2210		
Salt bridges							
Index	Residue	AA	Distance	Ligand group	Ligand atoms		
1	137A	LYS	5.44	Carboxylate	2369,2370

### Normal mode analysis in internal coordinates

**Fig. 7:**
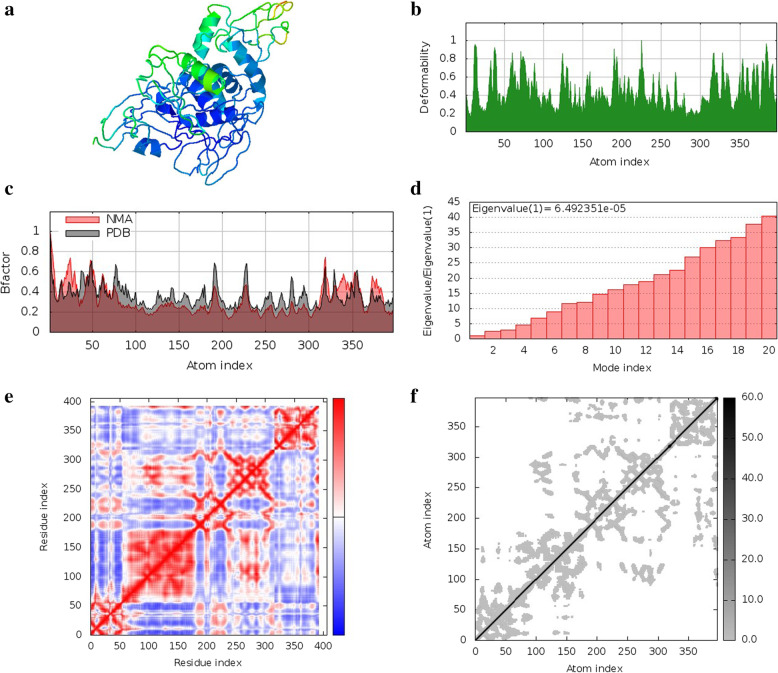
Normal mode analysis of quercetin 3-O-arabinoside 7-O-rhamnoside and papain-like protease complex **a** NMA mobility, **b** deformity, **c** B-factor, **d** eigenvalues, **e** covariance map, **f** elastic network of complex

**Fig. 8:**
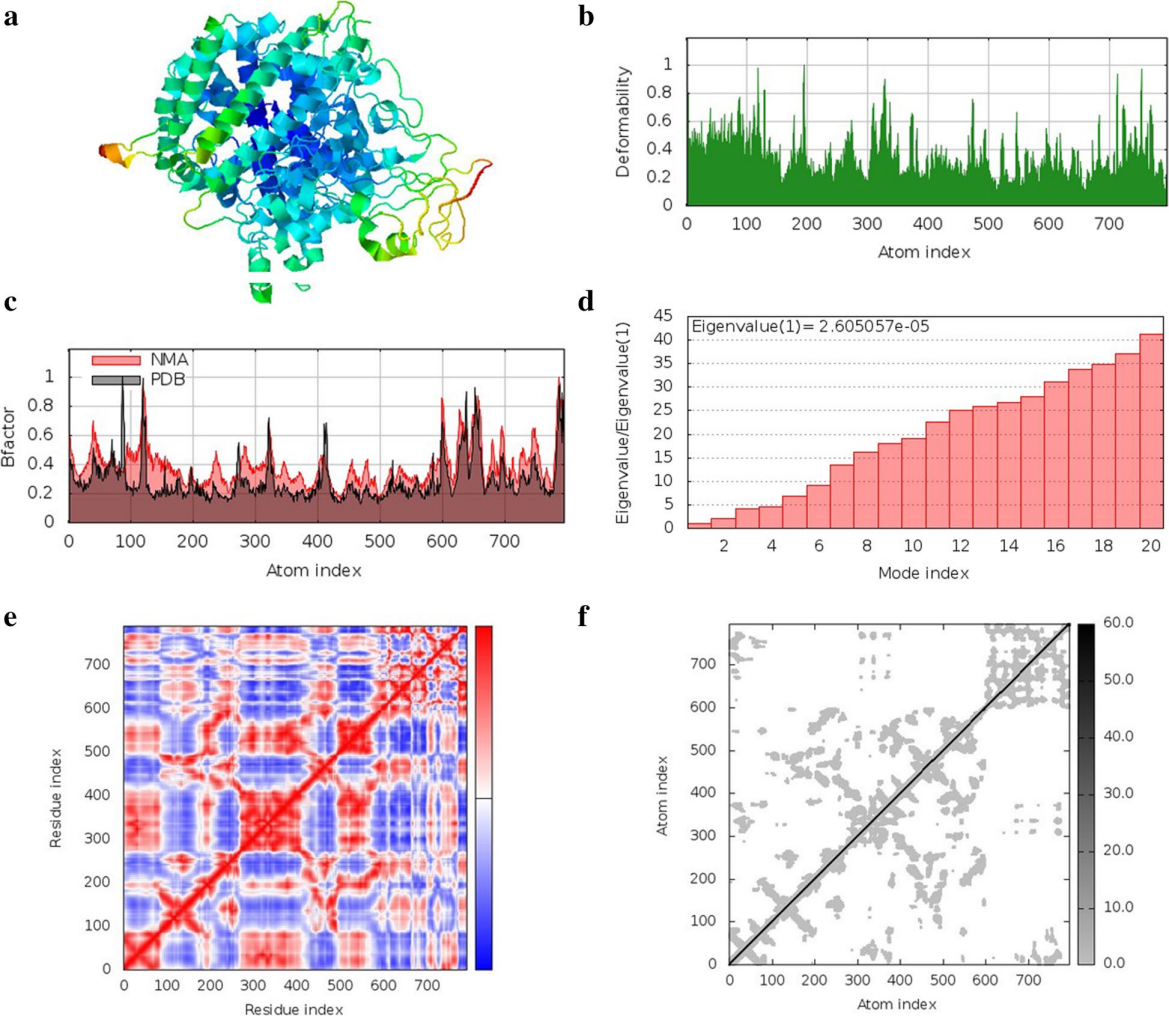
Normal mode analysis of quercetin 3-[rhamnosyl-(1- > 2)-alpha-L-arabinopyranoside] and spike protein receptor binding domain complex **a** NMA mobility, **b** deformity, **c** B-factor, **d** eigenvalues, **e** covariance map, **f** elastic network of complex

**Fig. 9:**
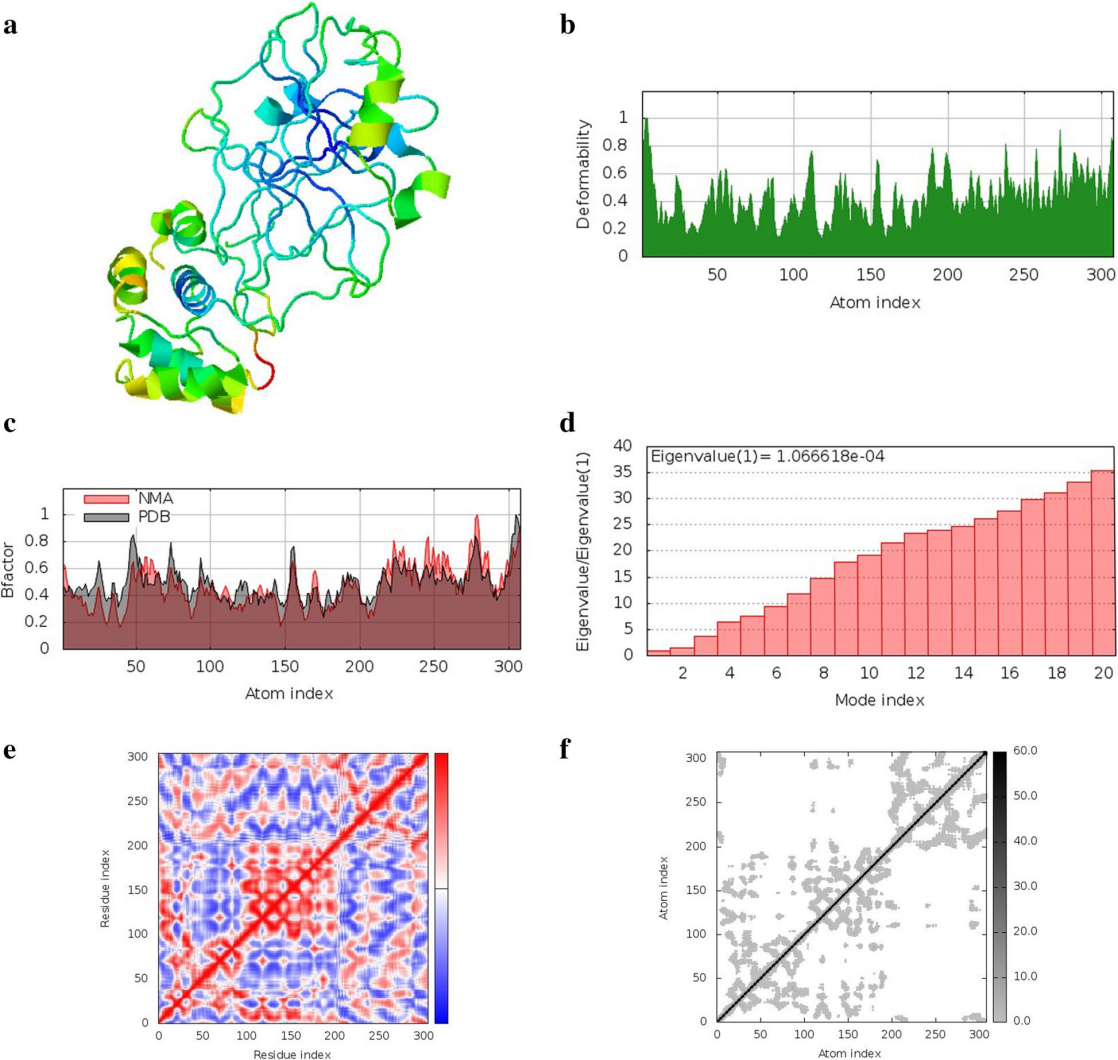
Normal mode analysis of quercetin-3-neohesperidoside-7-rhamnoside and 3C-like protease complex **a** NMA mobility, **b** deformity, **c** B-factor, **d** eigenvalues, **e** co-variance map, **f** elastic network of complex

Normal mode analysis in internal coordinates was performed using iMODS server to evaluate the movements of protein-ligand complexes. The NMA mobility of all the protein-ligand complexes is shown in Figs. 7a, 8a, and 9a. The main chain deformity is shown in Figs. 7b, 8b, and 9b, which shows hinges indicating high deformability regions. The B-factor values calculated by normal mode analysis are given in Figs. 7c, 8c, and 9c. Quantification of the uncertainty of each atom is calculated by B-factor values. Figures 7d, 8d, and 9d represent the eigenvalues of the complexes, which are a measure of the energy required for structure deformation. The lower the eigenvalue, the easier is the deformation. Eigenvalue for quercetin 3-O-arabinoside 7-O-rhamnoside and papain-like protease complex is 6.492351e−05. Eigenvalue for quercetin 3-[rhamnosyl-(1- > 2)-alpha-L-arabinopyranoside] and spike protein receptor-binding domain complex is 2.605057e−05 and the eigenvalue of quercetin-3-neohesperidoside-7-rhamnoside and 3C-like protease complex is 1.066618e−04. The covariance map, shown in Figs. 7e, 8e, and 9e, shows the coupling between pairs of residues. Correlated motion is represented in red, uncorrelated motion is represented in white, and anti-correlated motion is given in blue color. The elastic network of the structures, shown in Figs. 7f, 8f, and 9f, defines the pair of atoms connected by springs where each dot in the graph represents one spring between the corresponding pair of atoms. Darker grays indicate stiffer springs.

### Structural features of discovered bioactive compounds

**Fig. 10:**
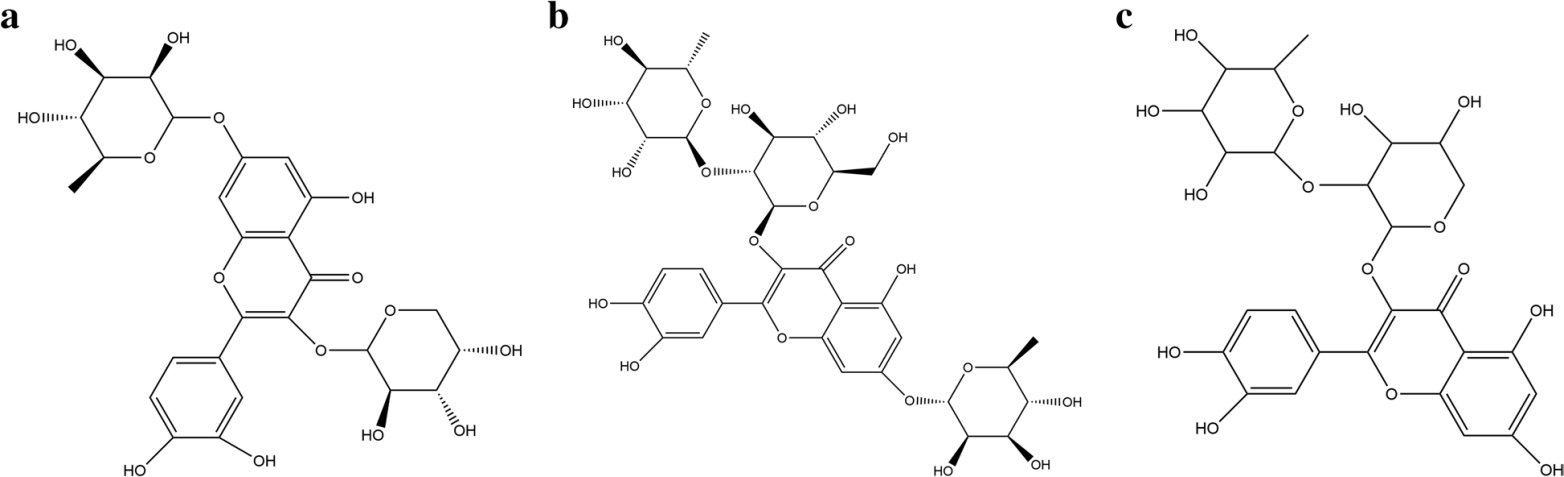
**a** Chemical structure of quercetin 3-O-arabinoside 7-O-rhamnoside. **b** Chemical structure of quercetin-3-neohesperidoside-7-rhamnoside. **c** Chemical structure of quercetin 3-[rhamnosyl-(1- > 2)-alpha-L-arabinopyranoside]

Quercetin 3-O-arabinoside 7-O-rhamnoside is chemically 2-(3,4-dihydroxyphenyl)-5-hydroxy-7-[(3R,4R,5R,6S)-3,4,5-trihydroxy-6-methyloxan-2-yl]oxy-3-[(3R,4S,5S)-3,4,5-trihydroxyoxan-2-yl]oxychromen-4-one. It is a quercetin O-glycoside, a trihydroxyflavone and a disaccharide derivative. It has a molecular weight of 580.14, XLogP3-AA value of **−** 0.9. The hydrogen bond donor count is 9, whereas the hydrogen bond acceptor count is 15. The rotatable bond count is 5, and the topological polar surface area is 245 **Å**^2^ (Fig. 10a).

Quercetin-3-neohesperidoside-7-rhamnoside is chemically 3-[(2*S*,3*R*,4*S*,5*S*,6*R*)-4,5-dihydroxy-6-(hydroxymethyl)-3-[(2*S*,3*R*,4*R*,5*R*,6*S*)-3,4,5-trihydroxy-6-methyloxan-2-yl]oxyoxan-2-yl]oxy-2-(3,4-dihydroxyphenyl)-5-hydroxy-7-[(2*S*,3*R*,4*R*,5*R*,6*S*)-3,4,5-trihydroxy-6-methyloxan-2-yl]oxychromen-4-one. It is a member of flavonoids and a glycoside having a molecular weight of 756.21, XLogP3-AA value of **−** 2. The hydrogen bond donor count is 12 whereas the hydrogen bond acceptor count is 20. The rotatable bond count is 8 and topological polar surface area is 324 **Å**^2^ (Fig. 10b).

Quercetin 3-[rhamnosyl-(1- > 2)-alpha-L-arabinopyranoside] is chemically 2-(3,4-dihydroxyphenyl)-3-[4,5-dihydroxy-3-(3,4,5-trihydroxy-6-methyloxan-2-yl)oxyoxan-2-yl]oxy-5,7-dihydroxychromen-4-one. It is a member of flavonoids and a glycoside having a molecular weight of 580.14, XLogP3-AA value of − 0.7. The hydrogen bond donor count is 9, whereas the hydrogen bond acceptor count is 15. The rotatable bond count is 5, and the topological polar surface area is 245 **Å**^2^ (Fig. 10c).

## Discussion

When it comes to SARS-CoV-2 structural proteins, the spike or S-protein is the most well-known, as it is the one responsible for the virus's attachment to the host cell. The S2 domain is responsible for viral fusion with the membrane of the host cell [33, 34]. The correct functioning of S protein will be disrupted if its attachment to the ACE2 receptor is prevented, its fusion function is inhibited, and the proteases responsible for its cleavage are inhibited [33]. 3CLpro is a coronavirus nonstructural protein. This enzyme cleaves viral polyproteins, resulting in the production of proteins necessary for virus replication and maturation. 3CLpro inhibition limits virus replication, making this protease a suitable therapeutic target [35]. PLpro can affect the innate immune response by cleaving ubiquitin and interferon-stimulated gene 15 (ISG15), recognized regulators of host innate immunity pathways, in addition to its protease action. Inhibition of this protease prevents viral replication [36].

Humayun et al. found different marine natural compounds to have a strong binding affinity for neuropilin-1 receptor of SARS-CoV-2. The molecular dynamics simulations also suggested the formation of stable complexes between the novel hits from natural marine compounds and neuropilin-1 receptor [37].

Ghosh et al. found that epigallocatechin-3-gallate (EGCG), epicatechin-gallate, and gallocatechin-3-gallate have strong binding affinity for Mpro and can hydrogen bond with one or both of its catalytic residues (His41 and Cys 145) in their investigation. In comparison to the unligated enzyme, produced complexes were more stable and less prone to conformational changes, as indicated by molecular dynamics (MD) simulations [38].

Herbacetin, rhoifolin, and pectolinarin are flavanoids that have previously been proven to be potent inhibitors of SARS-CoV Mpro. The IC50 values of the compounds were measured using a FRET-based assay and were 33.17, 27.45, and 37.78 M, respectively. They were projected to bind to the primary viral protease’s active site [39]. H herbacetin, pectolinarin, and baicalin were identified to block SARS-CoV-2 Mpro proteolytic activity [40].

Another promising natural medication against SARS-CoV-2 was discovered to be tannic acid. Mpro and the host cell protease TMPRSS2 are both inhibited by this polyphenol, which functions as a dual inhibitor. Tannic acid showed binding to Mpro with a dissociation constant of 1.1 M and TMPRSS2 with a dissociation constant of 1.77 M using surface plasmon resonance (SPR) [41].

In a recent in silico molecular docking research [38], EGCG, the major polyphenol in green tea, was identified as a possible inhibitor of SARS-CoV-2 Mpro [38].

The recent COVID-19 pandemic that caused severe necrosis and inflammation inside a host's body resulted in malfunctioning of supply of oxygen along with necessary nutrients into the host’s cells, proving to be a severe complication with subjects having compromised immunity. Therefore, in this current study, an effort was carried out to investigate the efficacy of quercetin derivatives against potential COVID-19 targets, i.e., papain-like protease, spike protein receptor-binding domain, and 3C-like protease with their combined immune modulation activity. Initially, the calculation of the drug-likeness score of individual molecules was done based on “Lipinski's rule of five” [42] because most of the drugs of plant origin are utilized via the oral route that identified 40 different compounds with high positive drug-likeness scores were considered to have good oral absorption (Table 1) that were used for further studies.

The concept of “single drug-single protein disease” involved in the regular drug discovery process might not be beneficial in managing the infectious disease. This is possible because of the greater affinity of the available pathogens (viruses and bacteria) to alter the multiple homeostatic functions of the protein molecules, which means different proteins present in pathogens are responsible for generating this effect. Management of this process can therefore be carried out by utilization of the “multi compound-multi protein-disease” concept, which is a modified drug development process interaction where multiple bioactives are involved in the regulation of multiple proteins [43], which in turn can be used as a basic key in the up-regulation of the immune system. Therefore, this present study deals with the combined synergistic phenomenon of quercetin derivatives, an investigation of which was done rather than the investigation of a single bioactive molecules to find out the multiple pathways that are directly or indirectly linked with the immune system.

The gene set enrichment analysis helped identify multiple pathways such as the p53 signaling pathway [44] and NF-kappa B signaling pathway [45] that has an involvement in upscaling of the immune system. Also, the other pathways like that of pathways in cancer, prostate cancer, MicroRNAs in cancer, hepatocellular carcinoma, endometrial cancer, breast cancer, and gastric cancer reflect quercetin derivatives potency in patients suffering from diseases like cancer from these mentioned pathways. Also, diseases like obesity and diabetes associated with pathways like p53 signaling pathways, PI3K-Akt, Wnt signaling are proven to be beneficial if regulated by the quercetin derivatives in patients with compromised immunity, thereby can act as a preventative strategy during the management of COVID-19. Further, herbal medicines rich in quercetin have potential antiviral properties against multiple viruses. Therefore, in this study, an attempt was conducted to evaluate the possible antiviral activity of quercetin derivatives against different viruses like influenza, HIV, rhinovirus, hepatitis B, hepatitis C, Trachoma, Picornavirus, CMV, and herpes virus based on their high-positive drug-likeness scores.

It was found that in the incorporation of viral polypeptides and deregulation of the homeostatic task of functional proteins, 3CL pro alters the ubiquitin regulatory protein consisting of 76 amino acids [46] that were majorly targeted by quercetin-3-neohesperidoside-7-rhamnoside. Furthermore, alteration of protein phosphate 1A and protein phosphate 1B, which regulates the replicase proteins to adjust viral cell life, is altered by PLpro [47] modulated by quercetin 3-O-arabinoside 7-O-rhamnoside. Similarly, the spike protein utilizes the ACE-2 (angiotensin-converting enzyme 2) as its target receptor to invade the host cell [48, 49], and this was chiefly modulation by quercetin 3-[rhamnosyl-(1- > 2)-alpha-L-arabinopyranoside]. In most of the studies conducted, the natural compounds were able to inhibit specifically one or two target proteases of SARS-CoV-2, but during our in silico study, we could identify three new hit derivatives of parent quercetin molecule, which could potentially inhibit all the three essential targets of SARS-CoV-2 as discussed above. Also, network pharmacology-based study and protein-protein interaction study were included along with molecular docking and molecular dynamics simulations to identify the specific pathways through which these potential quercetin derivatives will act, which was found to be missing from most of the in silico-based studies present in literature. The above results reflect the possibility of quercetin derivatives to act as a potential antiviral agent against SARS-CoV-2.

## Conclusion

The present study was carried out BY utilizing the in silico molecular docking tools to identify the affinity of quercetin derivatives binding against 3clpro, PLpro that was recorded previously. Also, the study was carried out to identify the affinity of quercetin binding against the spike protein receptor-binding domain. Quercetin 3-O-arabinoside 7-O-rhamnoside, quercetin 3-[rhamnosyl-(1->2)-alpha-L-arabinopyranoside], and quercetin-3-neohesperidoside-7-rhamnoside are considered as the lead hits. Also, the identification of the modulation of multiple pathways like p53, Wnt signaling pathway, RIG-I-like receptor signaling pathway was estimated using the network combined synergies generated. In addition, the quercetin derivatives were also found to be the modulators of specific disease pathways like diabetes and obesity, where immunity is compromised. All the available results provided a clear suggestion about the possible therapeutic activity in utilizing quercetin derivative as an immune modulator and an antiviral agent against the novel coronavirus. However, the above study’s findings are based only on the computer simulations, validation of which with an adequately designed experimental protocol is necessary.

## Future perspective and possible applications

The COVID-19 pandemic caused numerous social and economic disruptions around the world, and the effects of the epidemic are still being felt. Several efforts were made to counteract the effect and bring things back to normal. There is always a quest for lead compounds that can be useful in neutralizing the adverse effects of foreign substances entering our immune system, and the same is true for the COVID-19 therapy strategy.

In silico studies give a solid scientific foundation for three new quercetin derivatives as possible anti-SARS-CoV-2 agents. The in silico experiments indicated a substantial interaction of quercetin analogs with various SARS-CoV-2 proteases, leading to the conclusion that these newly identified quercetin derivatives could be used as a lead molecule. Although more research into the efficiency of three new quercetin derivatives is needed, it is possible that these analogs could be explored for antiviral therapy. It is possible to expand the current investigation to include in vitro and in vivo experiments using experimental animals to investigate the effects of quercetin analogs on antiviral therapy. It may be useful to confront SARS CoV-2 in a more substantial manner after acquiring positive results for the examined compounds using in vitro and in vivo procedures. This evidence-based study can be used to build a formulation of choice subject to achieving the intended effect, which will be useful against the COVID-19 therapy regimen. Furthermore, various developments in targeted delivery systems might be used in this lead molecule, which could be advantageous in delivering the agent of choice in the amount required to avoid future problems caused due to the virus strains.

## Data Availability

We declare that all the data generated are included in this study.

## Abbreviations

COVID-19: Coronavirus disease
ChEBI: Chemical Entities of Biological Interest
CMV: Cytomegalovirus
3CLpro: 3C-like protease
DENV-2: Dengue virus type 2
DIGEP-Pred: Prediction of drug-induced changes of gene expression profile
FRET: Fluorescence resonance energy transfer
HIV: Human immunodeficiency virus
HSV-1: Herpes simplex virus-1
NMA: Normal mode analysis
PLpro: Papain-like protease
Pa: Pharmacological activity
Pi: Pharmacological inactivity
RSV: Respiratory syncytial virus
SARS-CoV-2: Severe acute respiratory syndrome coronavirus 2
SMILE: Simplified Molecular Input Line Entry System
TMPRSS2: Transmembrane serine protease 2
UFF: Universal forcefield
